# Therapeutic potential of nitric oxide donors in the prevention and treatment of angiogenesis-inhibitor-induced hypertension

**DOI:** 10.1007/s10456-012-9327-4

**Published:** 2012-12-01

**Authors:** Peter Kruzliak, Gabriela Kovacova, Olga Pechanova

**Affiliations:** 1 Institute of Normal and Pathological Physiology, Centre of Excellence for Regulatory Role of Nitric Oxide in Civilization Diseases, Slovak Academy of Sciences, 813 71 Bratislava, Slovak Republic; 2 Department of Cardiovascular Diseases, International Clinical Research Centre, St. Anne′s Faculty Hospital, Masaryk University, Brno, Czech Republic; 3 5th, Department of Internal Medicine, University Hospital, Bratislava, Slovak Republic

**Keywords:** Angiogenesis, Vascular endothelial growth factor, Anti-angiogenic therapy, Nitric oxide donors, Cell proliferation

## Abstract

Angiogenesis is critical to tumor growth as well as to metastases. This process is tightly regulated by pro- and anti-angiogenic growth factors and their receptors. Some of these factors are highly specific for the endothelium—e.g., vascular endothelial growth factor (VEGF). A variety of drugs that target VEGF or its receptors have been developed for the treatment of different tumor types and a number of new agents is expected to be introduced within the coming years. However, clinical experience has revealed that inhibition of VEGF induces several side effects including hypertension and renal and cardiac toxicity. Angiogenesis-inhibitor-induced hypertension represents “crux medicorum” as it is often pharmacoresistant to antihypertensive therapy. We consider two most important pathomechanisms in the development of hypertension induced by angiogenesis inhibitors. The first represents direct inhibition of NO production leading to reduced vasodilatation and the second consists in increased proliferation of vascular medial cells mediated by NO deficiency and is resulting in fixation of hypertension. Based on the results of experimental and clinical studies as well as on our clinical experience, we assume that NO donors could be successfully used not only for the treatment of developed angiogenesis-inhibitor-induced hypertension but also for preventive effects. We thoroughly documented three clinical cases of cancer patients with resistant hypertension who on receiving NO donor treatment achieved target blood pressure level and a good clinical status.

## Introduction

Vessel formation occurs mainly through two sequential mechanisms: vasculogenesis—*de novo* formation of blood vessels during embryonic development, and angiogenesis—formation of new capillaries from preexisting vessels [1]. Angiogenesis is critical to tumor growth as well as to metastases [2, 3]. This process is tightly regulated by pro- and anti-angiogenic growth factors and their receptors. Some of these factors are highly specific for the endothelium (e.g., vascular endothelial growth factor—VEGF), while others have a wide range of activities in different cells (e.g., matrix metalloproteinases). A variety of physiologic and pathologic stimuli can induce production of angiogenic growth factors. Physiologic angiogenesis takes place during tissue growth and repair, during the female reproductive cycle, and during fetal development. In some diseases, the body loses the ability to control angiogenesis and new blood vessel growth is either excessive (e.g., cancer) or inadequate (e.g., coronary artery disease) [1–4].

As diseases relying on angiogenesis, such as cancer, are often partially driven by VEGF, inhibition of angiogenesis as a therapeutic strategy against malignancies was proposed by Folkman already in 1971 [5]. Meanwhile a variety of drugs that target endothelial growth factor or its receptors have been developed for the treatment of different tumor types and the expectation is that a number of new agents will be introduced within the coming years. VEGF receptors (VEGFRs) are expressed mainly on endothelial cells. As over 99 % of endothelial cells are quiescent under physiological conditions, it was expected that angiogenesis inhibition would have minimal side effects. However, clinical experience has revealed that inhibition of VEGF induces several side effects, including hypertension and renal and cardiac toxicity [6]. Insight into the pathophysiological mechanisms of these side effects is likely to contribute to improved management of the toxicities associated with VEGF inhibition. In this article we focus on the physiology of VEGF, on pathophysiological mechanisms of angiogenesis-inhibitor-induced hypertension and suggest a new hypothesis on prevention and treatment of several side effects of anti-angiogenic therapy.

### VEGF, VEGF-receptors and their role in angiogenesis

Vascular endothelial growth factor, a 45 kDa glycoprotein, is an angiogenic growth factor normally produced by endothelial cells, podocytes, macrophages, fibroblasts, and in malignancies by tumor cells or adjacent stroma. VEGF 165 (165 amino acids) is the predominant, biologically most active isoform and is referred to as VEGF in this review. The expression of VEGF is stimulated and regulated by multiple factors including hypoxia, which represents the main stimulator of VEGF transcription mediated through the hypoxia inducible factor 1 (HIF-1) [7, 8]. Transcription of the VEGF gene is inhibited by tumor necrosis factor alpha (TNF-α). VEGF upregulates the expression of endothelial nitric oxide synthase (eNOS) and increases nitric oxide production. Nitric oxide, on the contrary, may down-regulate VEGF expression via a negative feedback mechanism [9]. Tumor suppressor genes and oncogenes have also been found to play an important role in regulating VEGF gene expression. Loss or inactivation of tumor suppressor genes, such as von Hippel-Lindau (VHL), p53, p73, phosphatase and tensin homolog (PTEN) and p16, as well as activated forms of oncogenes, such as Ras, Src, human epidermal growth factor receptor 2 (HER2/neu) and breakpoint cluster region/Abelson (Bcr/Abl), increase VEGF gene expression [10].

Vascular endothelial growth factor binds two tyrosine kinase receptors, VEGF receptor 1 [VEGFR-1 or fms-like tyrosine kinase (Flt-1) murine homologue] and VEGF receptor 2 [VEGFR-2 or kinase domain region (KDR) human homologue or Flk-1 murine homolog]. Both receptors contain an extracellular region consisting of seven immunoglobulin-like domains, a hydrophobic transmembrane domain and a cytoplasmatic bipartite tyrosine kinase domain. VEGFR-1 and VEFGR-2 are expressed on endothelial cells of most blood vessels, including those of preglomerular, glomerular and peritubular vessels. Furthermore, these receptors are present on hematopoietic stem cells, circulating endothelial progenitor cells, dendritic cells, trophoblasts, monocytes, retinal progenitor cells and certain types of tumor cells [7, 11].

Most of the biologically relevant VEGF signaling in endothelial cells is mediated by VEGFR-2, activated by ligand-stimulated receptor dimerization and trans- (auto-) phosphorylation of the tyrosine residues in the cytoplasmatic domain [12]. The extracellular domain of Flt-1 is also present as a soluble protein (sFlt-1) that inhibits angiogenesis by forming an inactive complex with circulating VEGF [11]. VEGFR-1 has a 10-fold higher affinity for binding VEGF than VEGFR-2 but autophosphorylation of the tyrosine residues of the VEGFR-1 in response to VEGF binding is weak [11]. VEGFR-1, like sFlt-1, has been suggested to perform rather a decoy function by sequestering VEGF and leaving less VEGF available for VEGFR-2 than to mediate a mitogenic response [7]. VEGFR-3 (fms-like-tyrosine kinase (Flt)-4) is also a member of the receptor tyrosine kinases found mainly on lymphatic endothelium and is important for lymphangiogenesis [7, 10].

Vascular endothelial growth factor exerts a variety of biological activities. Its original name, vascular permeability factor, indicates that it enhances permeability. Furthermore, VEGF plays a key role in the mobilization of endothelial progenitor cells from the bone marrow, in endothelial cell proliferation, migration, survival and tube formation. It is a potent stimulator of angiogenesis during embryogenesis, menstrual cycle, wound healing and tumor growth. In addition, it inhibits antigen-presenting dendritic cells and stimulates monocyte chemotaxis and the expression of adhesion molecules. And finally, it induces vasodilation through activation of the nitric oxide pathway [7]. The above mentioned activities of VEGF operate via several pathways, including activation of the PI3 K/Akt (protein kinase B)/mTOR pathway, partly mediating VEGF-induced nitric oxide production via eNOS phosphorylation. Other actions include activation of phospholipase C-γ (PLC-γ), protein kinase C (PKC), Raf-1, extracellular-signal-regulated protein kinase (ERK1/2), focal adhesion kinase (FAK) and mitogen-activated protein kinase (MEK1/2) pathways [12–14].

Part of VEGF signaling occurs in a paracrine way, which is essential for the proliferation, survival, permeability responses and endothelial differentiation of the angiogenic cascade. An autocrine signaling loop (cell-autonomous) for VEGF is also required for survival of blood vessels. Both paracrine and autocrine activation are mediated by VEGFR-2 [15].

**Fig. 1:**
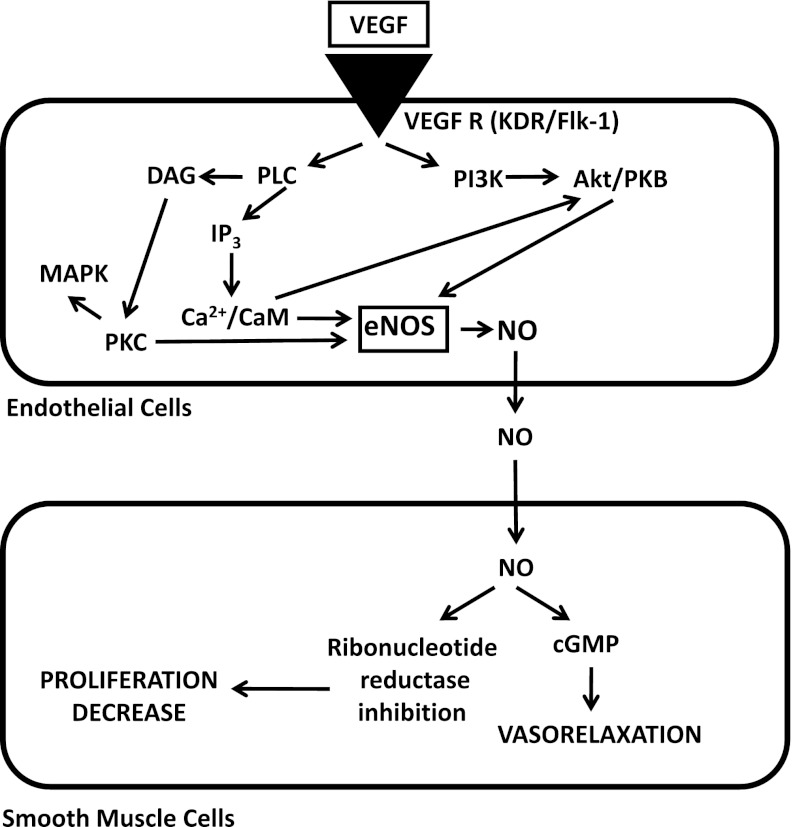
Proposed pathways for the VEGF-induced induction of NO synthesis in endothelial cells and the actions in smooth muscle cells. VEGF induces immediate NO synthesis through the PLC-Ca^2+^/CaM pathway and the induction of delayed NO synthesis implies Akt/PKB and PKC activition (eNOS upregulation). In smooth muscle cells, NO increases cGMP leading to vasorelaxation and inhibits ribonucleotide reductase resulting in decreased proliferation

Vascular endothelial growth factor has been shown to induce endothelium-dependent vasorelaxation in arteries of different sizes of various species, including human vessels [16–19]. This vasodilatation appears to be mainly mediated via nitric oxide, as it is attenuated in the presence of *N*-nitro-l-arginine (l-NNA), a nitric oxide synthase inhibitor [16–18]. Endothelium-dependent vasodilation is mainly due to VEGFR-2 stimulation [19]. In internal mammary arteries obtained from patients with severe coronary artery atherosclerosis, both PGI2 and nitric oxide appeared to contribute to VEGF-mediated vasorelaxation [18]. In addition to the observed vasorelaxation in vitro, in vivo experiments showed that intravenous injection of VEGF to conscious male Sprague–Dawley rats resulted in a dose-dependent decrease in mean arterial pressure and an increase in heart rate, almost immediately after VEGF injection [19]. In smooth muscle cells, nitric oxide may increase cGMP leading to direct vasorelaxation and inhibit ribonucleotide reductase resulting in decreased cell proliferation (Fig. 1).

### Angiogenesis inhibitors

A variety of drugs targetting VEGF or its receptors have been developed for the treatment of cancer, including monoclonal antibodies to VEGF, small receptor tyrosine kinase inhibitors (RTKIs) and circulating VEGF receptors to trap VEGF (‘VEGF-Trap’) [16]. Bevacizumab is a humanized monoclonal antibody that selectively binds VEGF and was the first FDA-approved VEGF inhibitor for systemic use in various forms of cancer including colorectal, breast, renal and nonsquamous, non-small cell lung cancer [20–22]. Bevacizumab has to be administered intravenously, in contrast to the RTKIs such as sunitinib (SU011248) and sorafenib (Bay 43-9006) which are suitable for oral administration. These agents are not selective as they target a number of tyrosine kinases. For instance, sunitinib inhibits VEGFR-1, VEGFR-2, VEGFR-3, platelet derived growth factor (PDGFR)-α and PDGFR-β, c-KIT, fms-like tyrosine kinase-3 (Flt3), colony stimulating factor receptor type 1 and the glial-cell-line-derived neutrophic factor receptor RET (rearranged during transfection). Sunitinib is approved for the treatment of imatinib-resistant metastatic gastrointestinal stromal tumors (GIST) and first-line treatment of metastatic renal cell carcinoma [23, 24]. Sorafenib is approved for the treatment of advanced renal cell carcinoma after failure of interleukin-2 or interferon-α treatment [23, 25]. The RTKIs are administered in cycles according to a 4 weeks “ON” and 2 weeks “OFF” (wash-out period) scheme. VEGFTrap (Regeneraon Pharmaceuticals, Tarrytown, New York, USA) is a protein consisting of portions of the extracellular domains of VEGFR-1 and VEGFR-2, fused to the Fc-portion of human immunoglobulin γ1. It binds and thereby inactivates VEGF in the circulation and tissues. The clinical effectiveness of this drug has still to be determined [25].

Contrary to expectations, inhibition of VEGF is associated with considerable cardiovascular and non-cardiovascular toxicity, hypertension, left ventricular dysfunction, cardiac ischemia, myocardial infarction, proteinuria, thyroid dysfunction, thrombosis, (cerebral) hemorrhage and skin manifestations, especially foot-hand syndrome.

### Hypertension Induced by therapy with angiogenesis inhibitors

The incidence of *de novo* or worsening hypertension in association with antiangiogenic therapy varies between 17 and 90 % [26, 27]. Hypertension has been reported in up to 36 % of patients during treatment with the humanized VEGF antibody bevacizumab with blood pressure normalization after treatment cessation [20, 28]. Initially reported incidences of sunitinib-induced hypertension varied from 16 to 23 %, but more recent studies reported an incidence of up to 47 % [29–32]. The incidence of hypertension was 67 % with the combined treatment of bevacizumab and sorafenib and 92 % with the combination of bevacizumab and sunitinib in patients with advanced solid tumors or renal cell carcinoma [33, 34]. Arterial hypertension in cancer patients can be associated with several complications, such as intracerebral hemorrhages, acute heart failure, and reversible posterior leukencephalopathy syndrome [30, 35, 36].

### Mechanisms of hypertension

Although the mechanism underlying the development of hypertension induced by angiogenesis inhibition still remains to be elucidated, decreased nitric oxide bioavailability is thought to be a critical factor. Results of the clinical study of Robinson et al. [37] have suggested that hypertension induced by VEGF inhibitors is mediated by suppression of NO production.

The eNOS is regulated also by the level of intracellular calcium. Intravascular calcium release by VEGF in vascular endothelial cells results from phospholipase C (PLC-γ) activation, which subsequently generates diacylglycerol (DAG) and activates inositol 1,4,5-triphosphate (IP3). IP3 induces the influx of calcium [38, 39]. The complex of calcium and calmodulin associates with eNOS to cause enzyme activation. The use of PLC inhibitors, CaM antagonists or intracellular calcium chelators attenuated Akt phosphorylation. Furthemore, the blockade of Ca/CaM-dependent Akt phosphorylation abrogated immediate NO production, whereas the inhibition of PI3K-dependent Akt phosphorylation was unrelated to immediate NO production. These data suggest that immediate NO synthesis requires the Ca/CaM-dependent Akt pathway [14]. Because eNOS is upregulated by VEGF, inhibition of VEGF by neutralizing antibodies or a VEGFR blocker leads to a decrease in nitric oxide production in endothelial cells that may account for the development of hypertension [23].

Hovens et al. [40] measured flow-mediated and nitroglycerin-mediated dilatation of the brachial artery as respective measures of endothelial-dependent and independent vasodilatation in patients treated with the experimental RTKI telatinib (Bay 57-9352). After 5 weeks of treatment, systolic and diastolic blood pressure were increased by respective 6.6 and 4.7 mmHg. This rise in blood pressure was associated with a small decrease in flow-mediated dilatation of 2.1 % and a decrease in nitroglycerin-mediated dilatation of 5.1 %. Although reduced nitric oxide availability might have caused the decrease in flow-mediated dilatation in this study, it cannot be ruled out that the development of hypertension itself had caused this reduction [40]. Of note, impaired nitric oxide production may not only cause a generalized vasoconstrictor response but may also affect renal sodium handling, contributing to the maintenance of hypertension in the longer run [41]. VEGF inhibitors might also lead to a reduction in the vascular density and adversely alter microcirculation. Although the effect of anti-VEGF drugs on nitric-oxide-dependent vasorelaxation is potentially reversible, the rarefaction of capillaries might not be so and bears therefore the risk of persisting hypertension [42, 43].

In essential hypertension, the activation of the renin–angiotensin–aldosterone (RAS) system plays a pivotal role and angiotensin-converting enzyme (ACE) inhibitors or angiotensin receptor blockers (ARBs) are commonly used for treatment. Whether the RAS is also activated in anti-VEGF-drug-induced arterial hypertension remains controversial [13]. Veronese et al. [44] reported that neither the renin-angiotensin system nor the sympathetic nervous system were involved in the development of this hypertension. Other factors influencing VEGF- inhibitors-induced hypertension include host susceptibilities, such as preexisting hypertension. Whether the type of cancer, and particularly renal cell cancer, is associated with a higher risk of anti-VEGF-induced hypertension remains controversial [45]. According to the results of our experimental studies, inhibition of nitric oxide synthase led to systemic hypertension with focal myocardial fibrosis, impaired arterial relaxation, and uncontrolled vascular medial proliferation attributed to the absence of smooth muscle cell proliferation inhibition by NO [46]. Mechanisms responsible for the antiproliferative effect of NO may involve cGMP-dependent and -independent phases acting at distinct points in the cell cycle, e.g., nitric oxide inhibits ribonucleotide reductase by cGMP-independent manner [47].

On the basis of experimental and clinical studies, we postulated the existence of at least two pathophysiological mechanisms leading to hypertension in patients treated with inhibitors of angiogenesis: (1) direct inhibition of NO production leading to reduced vasodilatation and increased vasoconstriction; (2) NO- deficiency-mediated increase in proliferation of vascular medial cells leading to the fixation of hypertension. Based on these two potential mechanisms, we assume that it would be possible to avoid hypertensive complications by using NO donors in patients treated with inhibitors of angiogenesis.

## Case reports

We present three case reports from our clinical practice corroborating the validity of our hypothesis.

Case No.1: A 67-year-old caucasian male was diagnosed of colorectal cancer with liver metastases without severe cardiovascular disease in history. He received chemotherapy, which consisted of a combination of 5-fluorouracil, leucovorin, irinotecan, and bevacizumab, an inhibitor of angiogenesis. After the second course of chemotherapy, hypertension developed in the patient. Blood pressure ranged from 180 to 210 mmHg systolic and from 100 to 120 mmHg diastolic. Despite comprehensive antihypertensive therapy based on administration of angiotensin converting enzyme inhibitors, thiazide diuretics, calcium channel blockers, beta-blockers and spironolactone, his blood pressure values ranged from 170 to 180 mmHg systolic and from 100 to 110 mmHg diastolic. Thereafter, the patient was treated with the NO donor molsidomine and we found a significant decrease in blood pressure values from 130 to 140 mm Hg systolic and 70 to 85 mmHg diastolic. This antihypertensive effect persisted over a long time following the seven months since introduction of the treatment.

Case No.2: A 59-year old caucasian male, a smoker, was treated for non-small cell lung cancer. In the past he had pulmonary artery embolism and was also treated for chronic gastritis. Due to cancer, left sided lobar lung resection was performed followed by adjuvant chemotherapy. Chemotherapy was based on a combination of cis-platin, vinorelbine and bevacizumab. Three weeks after the initiation of chemotherapy, hypertension resistant to antihypertensive treatment developed. Antihypertensive treatment was based on a combination of five drugs—angiotensin converting enzyme inhibitors, calcium channel blockers, beta blockers, thiazide diuretics and centrally acting hypotensives. Despite this therapy, systolic blood pressure was between 150 and 170 mmHg and diastolic from 95 to 110 mmHg. After addition of the NO donor isosorbide dinitrate, the clinical condition of the patient improved and his blood pressure decreased significantly to 130 mmHg systolic and 85 mmHg diastolic.

Case No. 3 was a 69-year old caucasian male with relapsed locally advanced renal cell carcinoma with metastases in the liver. After combined chemotherapy including avastin, he developed pharmacoresistant hypertension. Initially his blood pressure values ranged from 170 to 190 mmHg systolic and from 90 to 110 mmHg diastolic blood pressure, with currently administered combined antihypertensive therapy (ACE inhibitors, beta blockers, calcium channel blockers, thiazide diuretic, spironolactone). After adding isosorbide mononitrate to the treatment, a decrease of blood pressure to 130–140 mmHg systolic and 80–90 mmHg diastolic was monitored. The therapeutic response persisted even 6 months after treatment initiation.

## Conclusion

We assume two most important pathomechanisms operative in the development of hypertension induced by angiogenesis inhibitors. The first represents direct inhibition of NO production leading to reduced vasodilatation, and the second consists in the increased proliferation of vascular medial cells mediated by NO deficiency and resulting in fixation of hypertension. Moreover, NO deficiency may negatively affect the anticancer process itself. We used successfully NO donors in the treatment of hypertension in three cancer patients whose hypertension was a side effect of anti-angiogenic therapy and was resistant to the antihypertensive treatment administered. Based on the results of experimental and clinical studies as well as our clinical experience, we assume that the NO donors could be successfully used not only for the treatment of developed angiogenesis-inhibitor-induced hypertension but also as a preventive measure.

## Perspectives

Administration of NO donors before chemotherapy with angiogenesis inhibitors and/or during chemotherapy could significantly reduce the incidence and severity of this type of hypertension. The suggested approach may present a breakthrough in antihypertensive therapy focusing on pharmacotherapeutic prevention of hypertension in cancer patients receiving inhibitors of angiogenesis. Clinical studies are however needed to confirm the validity of this hypothesis.
